# The Role of Vitamin D in the Treatment of Carpal Tunnel Syndrome: Clinical and Electroneuromyographic Responses

**DOI:** 10.3390/nu16121947

**Published:** 2024-06-19

**Authors:** Antônio Vicente D. Andrade, Dallianny G. S. Martins, Gabriel S. Rocha, Gustavo S. Damasceno, Francisca T. S. Gomes, Yasmin P. F. Albuquerque, Paloma K. M. Melo, Marco A. M. Freire, Dayane P. Araújo, Lucidio C. Oliveira, Fausto P. Guzen, Paulo L. A. G. Morais, José R. L. P. Cavalcanti

**Affiliations:** 1 Laboratory of Experimental Neurology, Department of Biomedical Sciences, State University of Rio Grande do Norte, Mossoró, RN 59607-360, Brazil; antoniovicente@uern.br (A.V.D.A.); dalliannymartins@gmail.com (D.G.S.M.); gustavosouzad_11@outlook.com (G.S.D.); franciscatayna@alu.uern.br (F.T.S.G.); yasminpfernandes97@gmail.com (Y.P.F.A.); palomamelo@alu.uern.br (P.K.M.M.); dayanepessoa@gmail.com (D.P.A.); lucidioclebeson@uern.br (L.C.O.); faustoguzen@uern.br (F.P.G.); pauloleonardo87@hotmail.com (P.L.A.G.M.); 2 Behavioural and Evolutionary Neurobiology Laboratory, Federal University of Sergipe, Itabaiana, SE 49500-000, Brazil; gabriel.rocha9295@gmail.com (G.S.R.); freire.m@gmail.com (M.A.M.F.)

**Keywords:** carpal tunnel syndrome, vitamin D, supplementation, motor neuron, electroneuromyography

## Abstract

Carpal tunnel syndrome (CTS) is the most common cause of peripheral compressive neuropathy and consists of compression of the median nerve in the wrist. Although there are several etiologies, idiopathic is the most prevalent origin, and among the forms of treatment for CTS, conservative is the most indicated. However, despite the high prevalence in and impact of this syndrome on the healthcare system, there are still controversies regarding the best therapeutic approach for patients. Therefore, noting that some studies point to vitamin D deficiency as an independent risk factor, which increases the symptoms of the syndrome, this study evaluated the role of vitamin D supplementation and its influence on pain control, physical examination and response electroneuromyography to conservative treatment of carpal tunnel syndrome. For this, the sample consisted of 14 patients diagnosed with CTS and hypovitaminosis D, who were allocated into two groups. The control group received corticosteroid treatment, while the experimental group received corticosteroid treatment associated with vitamin D. Thus, from this study, it can be concluded that patients who received vitamin D, when compared to those who did not receive it, showed improvement in the degree of pain intensity, a reduction in symptom severity and an improvement in some electroneuromyographic parameters.

## 1. Introduction

Carpal tunnel syndrome (CTS) is an anatomical disorder that causes pain, numbness and tingling in the hand and arm of affected patients. Any condition that causes enlargement of the components of the carpal tunnel, reduces its cross-section or increases the pressure inside it can lead to symptomatic compression of the median nerve and is called CTS [1]. Risk factors for CTS include obesity, diabetes, monotonous wrist activity, pregnancy, genetic heredity and rheumatoid inflammation [2]. Patients with CTS tend to lose grip strength and manual functionality due to the pain caused by this condition, and people between 40 and 60 years old are the most susceptible to developing this pathology [3]. CTS is also more prevalent among women compared to men, afflicting more than twice as many women, at 88 per 100,000 men and 193 per 100,000 women [4].

Vitamin D is a fat-soluble vitamin and a steroid hormone involved in calcium and phosphorus homeostasis [5]. It is vital for the proper functioning of several body systems, such as the bone, cardiovascular, nervous, endocrine, immune and skin systems [6]. In this context, the individual with a lack of vitamin D is more vulnerable to diseases and this lack can trigger some clinical changes in all these systems, which can lead to infectious disease, metabolic syndromes and even cancer [7,8]. In addition to this, vitamin D has antioxidant and anti-inflammatory properties, which help maintain body homeostasis [9]. Therefore, low levels of vitamin D are associated with the development of diabetic neuropathy and some pain syndromes, pathologies that have a strong relationship with high levels of inflammation [10].

In addition, recent research has pointed to vitamin D deficiency as an independent risk factor that potentiates the symptoms of carpal tunnel syndrome, particularly pain and tingling [11,12]. In recent years, the role of vitamin D deficiency in the etiology of acute-chronic central and peripheral neurological diseases has been investigated, and the efficacy and treatment of vitamin D replacement have been demonstrated. However, few studies have been conducted to measure serum vitamin D levels in patients with CTS. Therefore, this study aims to evaluate the role of vitamin D and its influence on pain control as well as the electroneuromyographic response to conservative treatment of carpal tunnel syndrome.

## 2. Materials and Methods

This is a randomized, double-blind, prospective case series, seeking information on the role of vitamin D in the clinical and electroneuromyographic evolution of patients with carpal tunnel syndrome.

### 2.1. Ethics Statement

The research was submitted to and approved by the Ethics and Research Committee of the State University of Rio Grande do Norte ± UERN with opinion number 35457020.0.0000.5294, with the objective of ensuring the norms provided for in Resolution nº 466, of 12 December 2012, which considers respect for human dignity and special protection for participants in scientific research involving human beings.

It is emphasized that the present study respected the ethical aspects, especially the confidentiality, privacy and image-protection criteria of the participants. In order to avoid harm to the research participants, only the researchers could handle and access the questionnaires and information, in order to preserve the identity of each participant.

### 2.2. Study Sample

The sample initially consisted of 14 patients diagnosed with CTS treated at the orthopedics clinic of the College of Health Sciences (FACS) of the State University of Rio Grande do Norte (UERN) and at the Orthos Clinic in the city of Mossoró, Brazil. All patients were recruited voluntarily from pamphlets and advertisements on social networks and selected according to the inclusion and exclusion criteria.

### 2.3. Inclusion and Exclusion Criteria

The study included patients over 18 years old who were diagnosed with CTS, unilateral or bilateral, associated with hypovitaminosis D, and who had not used vitamin therapy in the last 6 months; patients who had not undergone previous surgery for the treatment of CTS; and patients who did not receive corticosteroid injection therapy.

Patients with systemic diseases that could compromise the results were excluded from the study, as were patients with other neuropathies or inflammatory syndromes already diagnosed; patients with a history of trauma to the limb with CTS; patients already treated surgically for CTS; and patients on dialysis and those with liver failure, coronary artery disease, decompensated diabetes mellitus, hypothyroidism, hyperparathyroidism and cervical hernia; and patients with obesity were also excluded from the sample of this initial study, in order to guarantee a better characterization of the electroneuromyography data.

### 2.4. Recruitment Plan

Initially, the research subjects were screened among individuals who voluntarily sought treatment and guidance for CTS at the orthopedics outpatient clinic. At first, the project was presented, as were the objectives, methods, questionnaire, medications, serum tests and electroneuromyography (ENMG) tests necessary at the beginning and end of the research. In addition, the risks and benefits were clarified, as were the ethical guarantees on the measures that ensure freedom of participation, the integrity of the participant and the preservation of data that may identify him, guaranteeing privacy, secrecy and confidentiality. All selected patients signed the Informed Consent Form. Patients who agreed to participate in the study were previously warned about the possibility of being treated only with conventional treatment and received clinical support even after the end of the research.

### 2.5. Biochemical Analyzes

Regarding the biochemical analyzes, the serum levels of 25-dihydroxyvitamin D were evaluated. The biological quantity for the analysis was 10 mL of venous blood, collected between 8 and 10 a.m. The researchers did not have access to the participants’ biological samples, only to the results. The collections were carried out by a nursing professional, with a qualification in clinical analysis, in the mentioned educational institutions, in a well-cleaned and comfortable environment for the collection. Analyses were carried out by a qualified analytical laboratory. In addition, participants received due guidance before the day of collection.

### 2.6. Electroneuromyography

All patients with CTS selected for the study underwent ENMG at the beginning and after the end of treatment to monitor cases during treatment. ENMG tests performed up to a maximum of 6 months before the beginning of the study were considered appropriate for the research. Such conventional sensory and motor conduction studies were performed by the neurologist. The median nerve was studied in both limbs with regard to sensory and motor conduction studies using a Medelec Synergy Electromyography machine (Clarity^®^). Filter settings used a 20–2000 Hz bandpass for the sensory nerve studies and a 2–10,000 Hz bandpass for the motor nerve studies. Sweep speed was set to 1 ms per division. Recordings from the bar electrodes were used for studies of motor and sensory nerves. A ground electrode was properly placed between the stimulation and recording electrodes. Nerve conduction studies were performed using standard techniques and guideline-based bipolar surface electrodes [10].

### 2.7. Physical Exam

The following tests were performed by the orthopedist before and after the end of treatment: Tinel’s sign and Phalen’s sign.

Tinel’s sign: If the patient feels paresthesia during manual percussion of the palmar surface of the wrist at the level of the median nerve, the test is considered positive. Sensitivity ranges from 26% to 79% and specificity ranges from 40% to 100% [13].

Phalen’s sign: If, during maximal active wrist flexion for one minute (elbow flexion), paresthesia appears in the area of the median nerve, the test is considered positive. In addition, there is a delay in the onset of symptoms by seconds. Sensitivity ranges from 67% to 83% and specificity ranges from 47% to 100% [13,14].

### 2.8. Questionnaire

A questionnaire was applied to all patients selected for the study, addressing the following issues: general data, anamnesis and physical examination and the Boston protocol for CTS.

The Visual Analog Scale (VAS) was included in the questionnaire and was used for all patients participating in the study, before, during and after the end of treatment, to assess pain intensity. The following criteria were used: 0–3, mild; 4–7, moderate; 8–10, severe [15]. The duration of symptoms and pain particularities were questioned in personal interviews.

The Boston protocol, known as the Boston Carpal Tunnel Questionnaire (BCTQ), is a questionnaire that was developed by Levine et al. [16], to be applied in patients with carpal tunnel syndrome, in order to assess the severity of the symptoms and the degree of manual skill. This assessment instrument was recognized as reproducible, valid and capable of responding to clinical changes in patients with CTS, and is mainly based on the quantification of paresthesia and pain in the hands and wrists, among other symptoms. In 2003, Campos et al. [17] proposed to translate it into Portuguese, and from then on, the questionnaire began to be widely used in our country. Its use allows standardization of subjective results and facilitates the comparison of symptoms and manual disabilities in scientific research and clinical trials [17,18].

### 2.9. Experimental Design of the Study

Randomized case series is a type of experimental study, in which participants are randomly placed into an intervention group or a control group. This is considered the gold standard among all clinical investigation methods used, as it is capable of producing direct scientific evidence with a lower probability of error to clarify a cause–effect relationship between two events. For randomization to work, investigators and participants must be unable to predict which group each participant will be allocated to—this is called allocation concealment; in addition, investigators must be unable to change the allocation of any participant after randomization.

After randomization, patients were allocated into two groups: G1 or Control Group and G2 or Experimental Group. The control group received conventional treatment for CTS, while the experimental group received conventional treatment + adjuvant (vitamin D). All patients received conventional conservative treatment for carpal tunnel syndrome, based on Chang et al. [19], which demonstrated that the use of oral corticoids for the conservative treatment of CTS was superior to the use of non-steroidal anti-inflammatory drugs.

Conventional treatment consists of using prednisone at doses of 20 mg in the first 10 days, 10 mg in the next 10 days, 5 mg in the next 20 days and 2.5 mg in the next 50 days, thus completing the treatment in 3 months. Group 1 received only conventional treatment, while Group 2, in addition to conventional treatment, received adjuvant treatment with vitamin D. According to the Brazilian Society of Endocrinology and Metabolism (SBEM) [19], doses for treatment vary according to the degree disability and the goal to be reached. However, in patients with hypovitaminosis D in general, weekly doses greater than 7000 IU (or greater than 1000 IU/day) are necessary to achieve 25(OH)D sufficiency. Therefore, following the SBEM recommendations, 2000 IU of vitamin D was administered per day, totaling 14,000 IU per week.

Both groups (G1 and G2) received the same guidelines and all professionals who had direct contact with the patients were not aware of which group they were allocated. Patients also did not have access to information about which group they belonged to.

### 2.10. Blinding Method

The preparation of formulations containing vitamin D and prednisone or just prednisone was carried out by a pharmacy not linked to laboratory members. “20 mg” of prednisone was used for the G1 capsules and “20 mg” of prednisone and 2000 IU of vitamin D for the G2 capsules. Both capsules looked the same and were contained in identical containers, so only the pharmacy staff knew what the corresponding formulations were.

Subjects included in the study were randomly allocated to receive treatment with prednisone alone or prednisone and vitamin D. The randomization plan in blocks of 4 was generated by a person who was not involved in any other phase of the research, through Microsoft Excel, using random number generation. Both researchers and patients were blinded in the study.

### 2.11. Statistical Analysis

Data were expressed as mean and standard deviation, as well as minimum and maximum values, simple frequency and percentage obtained through the statistical program SPSS (Statistical Package for the Social Sciences) version 23.0. To verify the association of the different categorical variables studied with the use of vitamin D, Fisher’s exact test was used. As for continuous variables, statistical differences within and between experimental groups (vitamin D and Control) for the times studied (before and after) were obtained after analysis of parametric assumptions, by paired *t* test and independent *t* test, respectively. When the Gaussian distribution was broken, Wilcoxon and Mann–Whitney were used. The established significance level was 5%.

## 3. Results

The final analysis was performed in 14 patients who were randomized in blocks to maintain group homogeneity (G1 and G2). Eight patients were allocated to G2 and received conservative treatment associated with vitamin D, while six patients belonged to the control group (G1) and received treatment with corticoid only. All participants were female, with an average age of 51 years (minimum 40 and maximum of 74 years).

The average height of the patients was 1.56 ± 8.0 and the weight in the first evaluation had an average of 71.78 kg ± 11.48. The value of vitamin D was 23.0 ± 4.85 ng/dL in the G2 group, while in the control group, it was 26.15 ± 2.43 ng/dL, which are values considered as hypovitaminosis based on the sample [20]. Of the fourteen patients with vitamin D deficiency included in the study, 12 (85.7%) had bilateral involvement and two (14.3%) had right upper limb involvement. Additionally, 78% had some associated comorbidity and used some kind of medication. Most patients (92.9%) were neither alcoholic nor a smoker. Of all patients, only 35% practiced physical activity on a regular basis.

**Figure 1:**
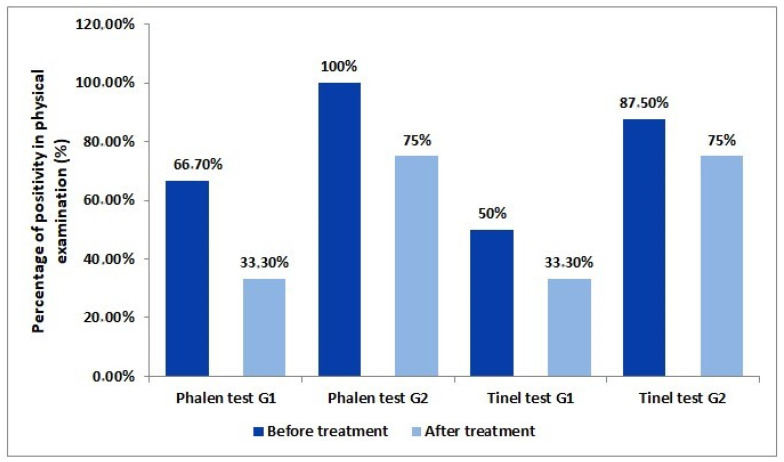
Variation in the percentage of positivity between the control group (G1) and the experimental group (G2) in the Phalen and Tinel tests before and after the three-month treatment.

In the evaluation of the physical examination, an 85.7% positivity rate for the Phalen test and a 71.4% positivity rate for Tinel was found. Additionally, 50% of G1 group patients initially presented a positive Tinel test. After 3 months of follow-up, 33.3% began to present a positive test. From the G2 group, 87.5% of patients had a positive Tinel test in the first consultation. After treatment, this number decrease to 75%. On the Phalen test, initially, 66.7% of the control group had a positive test. After 3 months, only 33.3% had a positive the test. From the intervention group, 100% presented a positive test at the first consultation. This number was reduced to 75% after 3 months of treatment (Figure 1).

According to VAS, the average pain score related to the two study groups was 7.43 ± 1.98 (minimum of 3 and maximum of 10). Nine patients were classified has having TCS in its severe form (64.3%), while four patients had a moderate form (28.6%) and one patient had a light form (7.1%).

**Figure 2:**
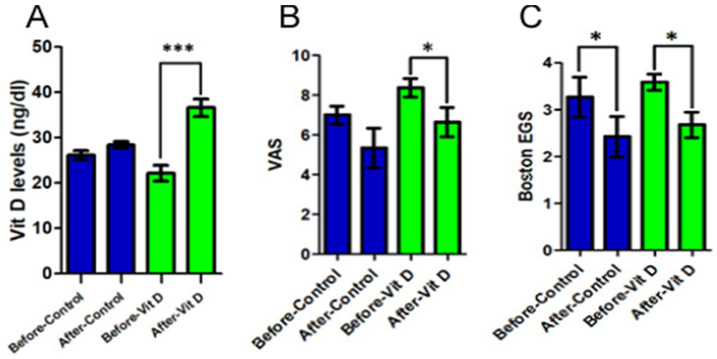
Comparison between control and experimental groups based on the effects of vitamin D supplementation. (**A**) After the use of vitamin D, Group 2 obtained a significant increase (*p* < 0.001) in plasma concentrations of this vitamin, while control group did not present changes in this aspect. (**B**) The group that supplemented with vitamin D showed a significant improvement (*p* < 0.05) in the visual analog scale; on the other hand, the control group showed no statistical difference. (**C**) When compared to each other, the groups did not show a significant difference (*p* > 0.05) after the Boston EGS intervention. Statistical significance was only found within each group separately. This means that the group that supplemented with vitamin D did not achieve a better result than the control group in this test. (*) *p* < 0.05 and (***) *p* < 0.001.

There was a statistically significant increase in the plasma concentration of vitamin D in the supplementation group (23.0 ± 4.85 to 36.31 ± 4.65; *p* < 0.001) (Figure 2A). In addition, this group also presented an improvement, with significance, of the graduation of the pain analyzed by VAS (8.38 ± 1.3 to 6.38 ± 1.85; *p* = 0.017), which was not detected in the control group (Figure 2B). Observing the results of the Boston scale to the EGS, there was a significant improvement in the gravity of symptoms in both groups (*p* < 0.026), which was not statistically reflected in the functional state score (*p* = 0.079 and 0.195), although it has presented a discreet tendency of improvement (Figure 2C).

After 3 months of intervention monitoring, we observed a statistically significant improvement in the motor latency of the median nerve (*p* = 0.001) and an improvement in the sensitive driving speed (*p* = 0.030). The other results, although they showed improvements related to other parameters, were not statistically significant in either group (*p* > 0.005).

## 4. Discussion

Our study observed a significant improvement in pain intensity and symptom severity in patients with CTS and vitamin D deficiency after 3 months of supplementation of the vitamin in question along with the initial standard treatment, demonstrated by changes in the VAS and Boston questionnaires (EGS). The findings were consistent with 21 vitamin D supplementation, when deficient, in patients with CTS [11,21,22].

Recently, some research has reported a potential link between the degree of CTS severity and serum vitamin D levels. Vitamin D deficiency is recognized as an independent risk factor for increased severity of CTS symptoms, particularly pain and tingling. The reason for this connection is that low levels of this vitamin can cause hyper-innervation and hypersensitivity in nerve fibers and increase the sensation and perception of pain in the pathological process [23]. These studies corroborate our result that patients with lower levels of vitamin D had a greater disease severity.

Regarding pain sensitivity, hypovitaminosis D is associated with nerve fiber hypersensitivity, coursing with persistent painful neuropathy [24]. Thus, studies suggest that vitamin D exerts its neuroprotective effect through the downregulation of L-type calcium channel expression or the upregulation of vitamin D receptor expression. In addition, vitamin D has antioxidant activity [25].

In addition to the effect on neurological function, the vitamin in question plays a role in the suppression of vascular endothelial growth factor, associated with increased inflammatory fibrosis, which may have a role in the triggering and clinical evolution of CTS. In this sense, although the literature points out that the Tinel and Phalen tests are used for diagnosis (due to their high sensitivity and specificity) and not for treatment control [26], our study showed that patients in both groups evolved with improvement in their responses to both tests. We suggest that due to the effectiveness of the tests, they could become very useful tools to assess clinical evolution in the face of conservative treatment, regardless of the use of vitamin D.

Preclinical experimental studies have shown that vitamin D acts on neuroprotective and neurotrophic functions and increases myelination and regeneration after peripheral nerve injury, which accelerates recovery and reduces neuronal damage [24,27]. Berridge [28] proposed that vitamin D acts by maintaining the integrity of cellular communication pathways. In addition to this, vitamin D also affects the process of neuronal plasticity, such as in axonal genesis.

Thus, for patients with low serum vitamin D levels, vitamin supplementation has been found to make an important contribution to the treatment of pain caused by compressive neuropathy in CTS [28,29]. In our study, the analysis performed using the Boston questionnaire showed that there was a statistically significant reduction in the severity of symptoms after 3 months of vitamin D supplementation. These results can be explained by the observation that vitamin D plays a role vital in the pain signaling pathway, supported by in vivo and in vitro studies. In this regard, studies have shown that vitamin D supplementation has benefits for relieving acute or chronic pain in other orthopedic conditions, including growing pain, low back pain and osteoarthritis.

Regardless of the specificity of the functional assessment instrument, the functional scores assessed in patients with CTS improved after vitamin D supplementation. However, the grip strength and the pinch strength, indirectly reflecting the muscles innervated by the median nerve, were not altered [24]. The research suggests that these different results may have been due to the very short period that vitamin D supplementation was given to affect motor functions [30]. In this sense, the symptom severity score assessed using the Boston questionnaire showed a statistically significant improvement. The functional status score, although it did not present the same result with statistical significance, showed a tendency towards improvement in the group supplemented with vitamin D. This can supposedly be explained by the short period of vitamin D supplementation.

Most of the patients included in previous experiments had CTS of mild to moderate severity and alterations in the latency of sensory conduction of the early median nerve when compared to motor conduction. On the other hand, the current experiment showed a higher prevalence of moderate to severe severity and did not show statistically significant changes in sensory conduction latency.

Regarding nerve conduction, current studies have shown that hypovitaminosis D is an important factor associated with decreased median nerve function. This function was evaluated taking into account sensory and motor conduction, with details of latency, amplitude and conduction speed [11].

In our study, the median nerve sensory conduction velocity results significantly improved after vitamin D supplementation. However, the motor conduction velocity was not visibly altered, which was in line with the unimproved grip and pinch strength. In this sense, all previously published studies showed the same result and suggested that a longer period of vitamin D administration could also influence the motor conduction speed [22,31].

Finally, analyzing the results and comparing them with those of previous studies, it was found that nerve conduction abnormalities can be evaluated using the parameters discussed above (ENMG testing and the VAS and Boston questionnaires) and help in diagnosis and treatment evaluation. Thus, future studies with larger numbers of participants, in addition to other possible evaluation methods and longer follow-up periods, are necessary to clarify the remaining doubts.

## 5. Conclusions

Our research showed that vitamin D supplementation improves the degree of pain severity in patients with CTS, which is analyzed using the VAS questionnaire; in addition, it influences the reduction in the severity of symptoms in patients with CTS, but it does not interfere with their functional status, factors that are both analyzed using the Boston questionnaire. Also, there was a significant improvement related to the Phalen and Tinel Tests, regardless of the use of vitamin D, suggesting that such tests can serve as useful tools for monitoring patients undergoing conservative treatment for CTS. In addition to this, vitamin D supplementation positively influenced motor conduction latency and sensory conduction velocity. The ENMG showed a statistically proven improvement in these two parameters mentioned above. Finally, although the study showed results with statistical relevance and that were consistent with what is found in the literature, studies with longer follow-up durations and higher doses (observing safety limits) that are inclusive of the various comorbidities that were excluded in this initial study should be encouraged to assess whether the effects of vitamin D supplementation in patients with CTS influence the long-term improvement of their functional results, since this result was not observed in the current study or in the literature.

## Data Availability

Data described in the manuscript, code book and analytic code may be made available upon request after an accepted proposal for a scientific work due to privacy.
